# Transcriptome Analysis Reveals Altered Expression of Memory and Neurotransmission Associated Genes in the REM Sleep Deprived Rat Brain

**DOI:** 10.3389/fnmol.2017.00067

**Published:** 2017-03-17

**Authors:** Santosh C. Narwade, Birendra N. Mallick, Deepti D. Deobagkar

**Affiliations:** ^1^Molecular Biology Research Laboratory, Center of Advanced Studies, Department of Zoology, Savitribai Phule Pune UniversityPune, India; ^2^School of Life Sciences, Jawaharlal Nehru UniversityNew Delhi, India; ^3^Bioinformatics Center, Savitribai Phule Pune UniversityPune, India

**Keywords:** REM sleep deprivation, transcriptome, brain excitability, epigenetics, histone code, memory consolidation, neurotransmitter

## Abstract

Sleep disorders are associated with cognitive impairment. Selective rapid eye movement sleep (REMS) deprivation (REMSD) alters several physiological processes and behaviors. By employing NGS platform we carried out transcriptomic analysis in brain samples of control rats and those exposed to REMSD. The expression of genes involved in chromatin assembly, methylation, learning, memory, regulation of synaptic transmission, neuronal plasticity and neurohypophysial hormone synthesis were altered. Increased transcription of BMP4, DBH and ATP1B2 genes after REMSD supports our earlier findings and hypothesis. Alteration in the transcripts encoding histone subtypes and important players in chromatin remodeling was observed. The mRNAs which transcribe neurotransmitters such as OXT, AVP, PMCH and LNPEP and two small non-coding RNAs, namely RMRP and BC1 were down regulated. At least some of these changes are likely to regulate REMS and may participate in the consequences of REMS loss. Thus, the findings of this study have identified key epigenetic regulators and neuronal plasticity genes associated to REMS and its loss. This analysis provides a background and opens up avenues for unraveling their specific roles in the complex behavioral network particularly in relation to sustained REMS-loss associated changes.

## Introduction

Sleep is amongst the most conserved instinctive behaviors. It has been classified into rapid eye movement sleep (REMS) and non-REMS. Disturbance including restriction, fragmentation or loss of REMS is associated with several acute as well as chronic psycho-somatic-behavioral dysfunctions (Van Dongen et al., 2003), cognitive and memory dysfunctions (Walker and Stickgold, 2014), neurodegenerative diseases, hypertension, diabetes, mood disorder, altered neuronal growth, development and excitability (Mallick and Singh, 2011). All these patho-physio-behavioral modulations can take place due to changes in the levels of one or more biomolecules or its metabolites or sometimes due to synthesis of new molecule(s). Although the precise function(s) of REMS and its detailed mechanism of regulation is not yet known, many hypotheses have been put forward about its role in maintaining several normal physiological processes (Stickgold et al., 2001). As a unified hypothesis it has recently been proposed that REMS serves house-keeping functions by maintaining brain neuronal excitability (Mallick and Singh, 2011). Its functions have been investigated by evaluating the effects of rapid eye movement sleep deprivation (REMSD) on several physiological parameters. However, regardless of the advances in our knowledge on regulation and function of REMS (Mallick et al., 2011), there are serious lacunae in our understanding of the molecular mechanism underlying REMS-loss associated alteration of physiological conditions and symptoms, particularly its sustained long-term effects (Mehta et al., 2015).

Rapid eye movement sleep deprivation affects several hormones, metabolites (Spiegel et al., 1999; Zalfa et al., 2006), interleukins, enzymes, neuronal structural proteins and induces apoptosis (Biswas et al., 2006) in the brain; however, transcriptional regulation of these processes is unknown. In order to understand the long term effects of REMSD, including expression of symptoms due to chronic REMS-loss and associated disorders, it will be useful to examine the modulation of bio-molecules which is likely to occur as a consequence of altered gene expression. Microarray based analysis of brain samples upon total sleep loss, which includes loss of both REMS as well as non-REMS, has been reported (Cirelli et al., 2004; Vecsey et al., 2012). Short-term (8 h) total sleep loss affected transcription of about 5% of cortical genes (Cirelli et al., 2004), while long-term total sleep deprivation significantly induced expression of transcripts coding for many proteins including those related to synaptic plasticity, memory consolidation, membrane trafficking (Tononi and Cirelli, 2014), mitochondrial proteins, heat-shock proteins and synaptic potentiation (Ravassard et al., 2009). In this study, we have employed next generation sequencing (NGS) technology to analyze the brain transcriptome (RNA seq) of control and selective REMS deprived rats. Our findings provide insight into the possible molecular-genetic mechanisms in the brain that are closely associated with pathological conditions due to REMS loss in particular and sleep disorder in general.

## Materials and Methods

### Animal Care and Rapid Eye Movement Sleep Deprivation

The experiments were carried out on male Wistar rats (225–250 gm) maintained at 24 + 2°C and 12:12 h light:dark cycle with *ad libitum* free access to food and water. Experimental procedures were approved by the Institutional Animal Ethical Committees of the Savitribai Phule Pune University, Pune and Jawaharlal Nehru University, New Delhi in accordance with the national guidelines provided by the Committee for the Purpose of Control and Supervision on Experiments on Animals (CPCSEA). Home cage normal rats were taken as free moving control. Standard protocol was followed for REMSD by the flower pot method (Gulyani and Mallick, 1993, 1995; McDermott et al., 2003). In brief, for REMSD the experimental rat was maintained on a small platform (6.5 cm diameter) surrounded by water for 4 days (96 h) (Gulyani and Mallick, 1993; Amar and Mallick, 2015). Rats were deprived of REMS for 4 days as we have shown that the period is optimum for inducing change in many biomolecules (Mallick and Singh, 2011). The rats become aggressive and irritated, humans also become irritated after sleep-loss. To rule out the effects due to non-specific factors, another control rat was maintained on larger platform of 13 cm diameter surrounded by water. These large platform control rats were maintained under similar conditions as that of the experimental (REMSD) rats except for the platform size. They experienced non-REMS as well as REMS comparable to free moving control. Recovery control rats were deprived of REMS for 4 days (96 h) and then allowed to recover from REMS loss for 3 days in home cages. In yet another group, the rats were REMS deprived for 4 days and while deprivation was continuing, on the third and fourth day of deprivation the rats received (0.5 ml of 4 mg/kg i.p.) alpha1 adrenoceptor (AR) antagonist, Prazosin (PRZ) dissolved in 20% *N,N*-dimethylacetamide (*N,N*-DA). Prazosin was dissolved in a very small (0.1 ml) quantity of *N,N*-DA and made to 0.5 ml with saline for i.p. injection. In our earlier studies, the same concentration (20%) of *N,N*-DA as vehicle was ineffective on sleep-waking as well as thermoregulation when injected (0.4 or 0.2 μl) locally directly into specific sites in the brain (Mallick and Alam, 1992; Mallick and Joseph, 1998; Pal and Mallick, 2006) or injected (0.5 ml) i.p. (Jaiswal and Mallick, 2009); therefore, we did not consider necessary to carry out separate *N,N*-DA treated control group(s). The Prazosin blocks REMSD-associated noradrenaline (NA)-induced increase in Na–K ATPase activity (Gulyani and Mallick, 1995). After completion of REMSD, the rats were euthanized, decapitated and the brains taken out for further processing.

### Synaptosomal Sodium Potassium ATPase Enzyme Assay

#### Synaptosome Preparation

The synaptosomes were prepared from all the control and experimental (REMSD, large platform control, free moving control, recovery control and Prazosin treated) rat brains (*n* = 4 rats per group) as described earlier (Gulyani and Mallick, 1995). Briefly, the brains were quickly removed and homogenized in 10 ml chilled buffer containing 12 mM Tris, 0.32 M sucrose and 1 mM EDTA, pH 7.4. The homogenate was centrifuged at 6000 rpm (3000 × *g*) for 5 min and then 5 ml of the supernatant was centrifuged at 12,000 rpm (11000 × *g*) for 20 min. The pellet was re-suspended in 1 ml of homogenizing buffer, which was then loaded on to 1.2 M and 0.8 M sucrose gradient and ultracentrifuged in a swing-out rotor for 2 h at 25,000 rpm (105,000 × *g*). At the end of ultracentrifugation the synaptosomes were obtained as a band at the interface of the sucrose gradients. The band of synaptosomes was retrieved, diluted with 2 ml homogenizing buffer and centrifuged for 45 min at 25,000 rpm (105,000 × *g*) to obtain the synaptosomes as a pellet. All the steps were carried out at 4°C.

#### Sodium Potassium ATPase Activity

The ouabain sensitive Na–K ATPase activity was estimated following the method of Akagawa and Tsukada with minor modification as reported earlier (Gulyani and Mallick, 1995). In brief, 30 μg of the synaptosomes were incubated at 37°C for 15 min in the reaction buffer containing 100 mM NaCl, 20 mM KCl, 5 mM MgCl_2_, 3 mM ATP and 50 mM Tris, pH 7.4. Ouabain (1 mM) was used as Na–K ATPase specific blocker and ATP was used as the substrate. The liberated inorganic phosphate (Pi) was estimated spectrophotometrically using Fiske and Subbarow (1925) method. The Na–K ATPase activity was calculated as the difference of activities in the sample in presence and absence of ouabain and expressed as μmoles of Pi released per mg protein per hour.

### RNA Extraction and Sample Preparation

For the extraction of total RNA, the REMSD and free moving control whole brains were individually homogenized in TRIzol (Life Technologies, USA) using mortar and pestle according to the protocol provided by the manufacturer. Total RNA was treated with TURBO DNA free kit (Life Technologies, USA) to remove DNA contamination. Qubit RNA BR Assay Kit (Life Technologies, USA) was used for quantitation of RNA concentration and Agilent Bioanalyzer 2100 (Agilent, Santa Clara, CA, USA) was used for quality assessment of each sample. All samples with RNA integrity number (RIN) above 7.4 were taken for further analysis.

### Construction of RNA-seq Libraries, RNA Sequencing, and Data Generation

The mRNA sample preparation was done by using Truseq mRNA sample preparation kit (Illumina, San Diego, CA, USA) as per manufacturer’s instructions. RNA was treated with Ribo-Zero magnetic kit (Epicentre, USA) for rRNA depletion in accordance with manufacture’s protocol. The poly (A) RNA was captured from rRNA depleted RNA using poly-dT magnetic beads and was chemically fragmented to a size around 200–300 bp. These RNA fragments were used for first and second strand cDNA synthesis. Single (A) base overhang was added to the blunt ends to enable adapter ligation with the (T) base overhangs. These product fragments were purified and used for enrichment by using adapter specific PCR. The libraries were amplified using c-Bot (Illumina, San Diego, CA, USA) to produce clusters. By using Agilent Bioanalyzer quality and size distribution of the cDNA library was checked. Fragments size for the cDNA library were between 200 and 500 bp, with a peak at ∼300 bp. Qubit 2.0 Fluorometer (Life Technologies, Foster City, CA, USA) were used for the quantification of libraries. The cDNA library was sequenced by 100 × 2 paired end on Illumina Hiseq 2000 sequencer (Illumina, San Diego, CA, USA).

### Read Mapping and Expression Analysis

Based on the quality of sequence reads, they were trimmed where necessary to retain only high quality sequence for further analysis. From the trimmed paired-end reads the unwanted sequences, e.g., the ribosomal RNAs, transfer RNAs, mitochondrial genome sequence, adapter sequences and others were considered as contamination and removed using bowtie2 (version 2.1.0), in-house Perl scripts and picard tools (version 1.85). The paired end files so generated were trimmed using SolexaQA with Phred quality score of 30. Adapter trimming and length sorting were performed using trimmomatic PE software package. The pre-processed reads were aligned to the reference *Rattus norvegicus* genome and gene model downloaded from UCSC database (ASSEMBLY NAME: Rnor_6.0; ASSEMBLY DATE: 1 July 2014). The alignment was performed using TopHat program (version 2.0.8) with default parameters. We only used uniquely mapped reads for further analysis. The alignments were processed and the expression levels were quantitated using Cufflinks based on the gene loci annotations and GENCODE transcripts (Rajpathak et al., 2014). The reads mapping for the corresponding gene annotations were calculated as fragments per kilo base of transcript per million mapped reads (FPKM).

In order to identify differentially expressed genes (DEGs), RNA sequencing data was analyzed using Cuffdiff with a *P*-value < 0.05 in log-transformed expression value (log_2_ FPKM). All genes with significant expression change (*P*-value < 0.05) were further analyzed (Yu et al., 2014). Also, DEGs with more stringent false discovery rate (FDR) with the *q-*value set at 0.05 were analyzed separately.

### Functional Analysis of Differentially Expressed Genes

The Database for Annotation, Visualization and Integrated Discovery (DAVID) was used to identify significantly enriched Gene Ontology categories and for functional annotations of DEGs. Functional annotation clustering was carried out for ranking enriched term groups with enrichment score (ES) ≥ 1.3 and *P* < 0.05. Groups with higher ES have been suggested to be more relevant to the study.

### Quantitative Real Time PCR (qRT-PCR) Assay

Validation of RNA-Seq data was carried out by qRT-PCR of 13 DEGs. RNA was isolated separately from the REMSD, large platform control, free moving control, recovery control and Prazosin treated rat brains (*n* = 5 rats per group) using TRIzol as described above. The cDNA was prepared using 1 μg of total input RNA from all samples using Superscript III (Invitrogen, USA) according to the manufacturer’s instruction. qRT- PCR was carried out using SYBR Green master mix (TAKARA, USA) for detection in StepOnePlus Real Time-PCR system (ABI, USA). We evaluated relative expression of each gene against the expression of GAPDH gene as internal control in respective brain sample. The primer sequences have been shown in **Table 1**. Differences in gene expression were statistically evaluated using one-way analysis of variance with Neuman–Keul’s test; at least *P* < 0.05 was taken as statistically significant.

**Table 1:** Lists of primers used in gene expression analyses by real-time qPCR.

Gene	Forward primer (5′–3′)	Reverse primer (5′–3′)	Acc number
OXT	GCTGCGCTAGACCTGGATATG	GAAGCAGCCCAGCTCGT	NM_012996
GAPDH	GGGAAACCCATCACCATCTT	CCAGTAGACTCCACGACATACT	NM_017008.4
AVP	CGCCATGATGCTCAACACTA	CTTGGGCAGTTCTGGAAGTAG	NM_016992.2
LNPEP	TTAGCCTACATCCAAACCTAACC	GGAGAATGATGTCCCGTGTATC	NM_001113403.1
RMRP	CTGAGTGCTCGTCACTCTCT	GCTCTCTGGGAACTCACCT	NR_002703.1
HIST2H4	GCAGAGGAAAGGGTGGTAAG	TGGATGTTATCCCGCAAGAC	NM_001123469.1
GRIN2B	GCTCAGATCCTCGACTTCATTT	ACTCATCCTTATCCGCCATTATC	NM_012574.1
HCRT	GCATCCTCACTCTGGGAAAG	GGTTACCGTTGGCCTGAA	NM_013179.2
PMCH	CAGGAACGTAGAAGACGACATAG	TCCAGAGAAGGAGCAACAAC	NM_012625.1
ATP1B2	TGGTTGAGGAGTGGAAGGA	CGAGGTAGAAGAGGAGGATGAA	NM_012507.3
DBH	GGTGAACAGAGACAACCACTAC	GCACGAAGTGATGAGGACAT	NM_013158.2
NPAS4	CCTAATCTACCTGGGCTTTGAG	GGCGGTAGTGTTGAGAAGAA	NM_153626.1
EGR1	GCTCACTCCACTATCCACTATC	GTTGGGACTGGTAGGTGTTATT	NM_012551.2
SYT2	CTGACTGAAGGAGAAGGAGAAG	CTGGTTGGCTTGGAAATCATAG	NM_012665.1

*OXT – oxytocin; GAPDH – glyceraldehyde 3-phosphate dehydrogenase; AVP – arginine vasopressin; LNPEP – leucyl/cystinyl aminopeptidase; RMRP – RNA component of mitochondrial RNA processing endoribonuclease; HIST2H4 – histone cluster 2, H4; GRIN2B – glutamate receptor, ionotropic, *N*-methyl D-aspartate 2B; HCRT – hypocretin (orexin); PMCH – pro-melanin concentrating hormone; ATP1B2 – Na–K ATPase beta-2 polypeptide; DBH – dopamine beta-hydroxylase; NPAS4 – neuronal PAS domain protein 4; EGR1 – early growth response 1 and SYT2 – synaptotagmin 2.*

## Results

### Changes in Na–K ATPase Activity after REMSD

**FIGURE 1:**
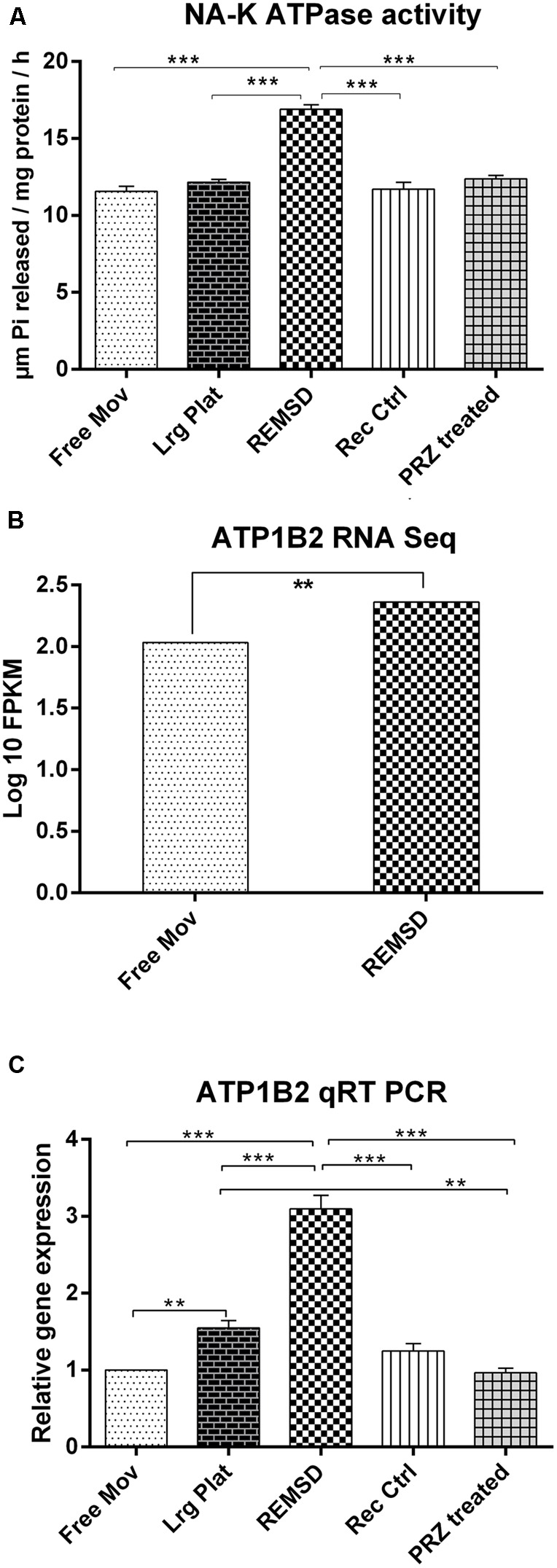
**Na–K ATPase activity and expression: (A)** Na–K ATPase activity in synaptosomes prepared from rat brains (*n* = 4 rats per groups). **(B)** RNA seq data showing significant up-regulation of ATP1B2 transcripts upon REMSD (*P* < 0.019) as compared to Free Mov rat brain. **(C)** These results were confirmed by qRT-PCR (*n* = 5 rats per groups). Statistical analyses were performed using one-way ANOVA followed by Newman–Keuls multiple comparison tests. Data are expressed as the mean ± SEM (^∗∗^*P* < 0.005, ^∗∗∗^*P* < 0.001). REMSD – rapid eye movement sleep deprivation; Free Mov – free moving control; Lrg Plat – large platform control; Rec Ctrl – recovery control; and PRZ treated – Prazosin treated group.

It has been consistently shown that the synaptosomal Na–K ATPase activity is increased after REMSD in rats. We have estimated Na–K ATPase activity in control and REMS deprived rat brains as circumstantial and associated confirmation of REMSD. Indeed we observed that the enzyme activity increased significantly upon REMSD as compared to free moving control (*P* < 0.001) and large platform control (*P* < 0.001). The activity returned to the baseline level upon 3 days of recovery sleep after REMSD or after Prazosin treatment to the REMS deprived rats (**Figure 1A**). Transcriptome analysis showed that ATP1B2 transcripts (responsible for synthesis of Na–K ATPase) were significantly up-regulated (*P* = 0.019) upon REMSD (**Figure 1B**). This was further confirmed by qRT-PCR where a significant (*P* < 0.001) increase in the level of mRNA of ATP1B2 was observed in REMSD rat brains (**Figure 1C**) as compared with free moving control, large platform control, recovery control and Prazosin treated rats. The ATP1B2 transcripts were also elevated in the large platform control rat brains as compared to that of the free moving control (*P* = 0.007) and Prazosin treated (*P* = 0.005) rats; however, it was comparable to recovery control rats.

### Rat Transcriptome Data Generation and Analysis

Poly-A tailed RNA was obtained from the REMSD and free moving control rat brains. RNA sequence reads were generated using paired-end sequencing method. A total of 31.34 and 59.31 million raw sequence reads from REMSD and free moving control rat brains, respectively, were generated using Illumina Hiseq 2000 (Illumina, USA). After quality filtering (≥30 Phred score) about 90.6% and 88.4% of the reads from the REMSD and the free moving control, were used for analysis. Out of the analyzed reads, 91% of the sequence reads were mapped back to the reference *Rattus norvegicus* genome (ASSEMBLY NAME: Rnor_6.0; ASSEMBLY DATE: 1 July 2014). RNA sequencing raw data was generated and the results of alignment have been summarized in **Table 2**. The FPKM values of each gene were calculated using Cufflink and raw data have been submitted to NCBI SRA database with accession number SRR3270972 (free moving control) and SRR3318878 (REMSD).

**Table 2:** Sequencing raw data generated and data analysis results.

Sample name	Raw reads (paired end)	Base (Gb)	GC (%)	% of data ≥ Q30	Raw read length (bp)
**A) Fastq File Summary:**
REMSD	15,673,204	3.1	49.67	90.6	100 x 2
Free Mov	29,657,987	5.9	48.8	88.4	100 x 2

**B) Read alignment summary:**

**Sample name**	**Total reads**	**Aligned read count**	**Aligned %**

REMSD	31,346,408	25,591,827	91.36
Free Mov	59,315,974	45,404,529	91.68

***(A)** Fastq file summary after RNA seq data generation, about 90% data passes Phred score ≥ Q30 and **(B)** read alignment results after data analysis, around 91% of data aligned to *Rattus norvegicus* genome for both REMSD and Free moving control (Free Mov).*

### Differentially Expressed Genes (DEGs) and Functional Analysis

**FIGURE 2:**
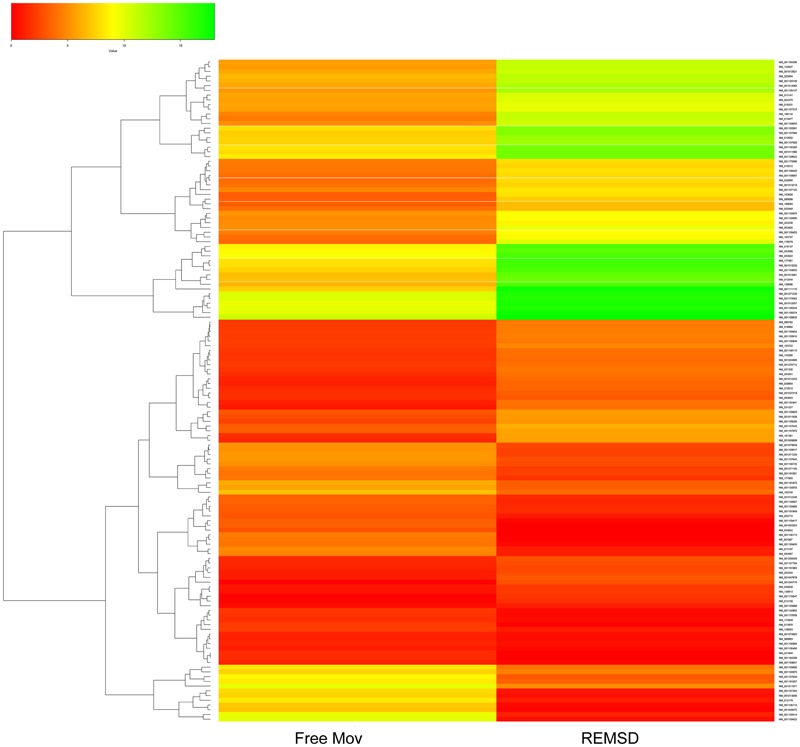
**Heat Map of differentially expressed genes (DEGs).** Clustered heat Map depicting DEGs after 96 h of selective REMSD as compared with Free Mov control. A total of 216 genes showing significantly altered gene expression (*P* < 0.05); out of which 80 genes were up-regulated and 136 genes were down-regulated upon REMSD.

Cuffdiff analysis and quantile normalization showed that 216 genes were (*P* < 0.05) differentially expressed; 136 were up-regulated, while 80 were down-regulated after REMSD as shown in the heat-map (**Figure 2**). Functional analysis of the gene-sets showing differential expression (*P* < 0.05) revealed enrichment of several functional categories as shown in Supplementary Tables S1, S2. The gene ontology of these clustered categories represents genes involved in chromatin assembly (ES = 3.25; *P* < 0.0000046), regulation of synaptic transmission (ES = 1.49; *P* < 0.0032), methylation (ES = 3.25; *P* < 0.000017), behavior (ES = 1.49; *P* < 0.000051), regulation of homeostatic processes (ES = 2.30; *P* < 0.0008), transcription regulation (ES = 2.03; *P* < 0.00013), regulation of neurological system process (ES = 1.49; *P* < 0.0006), regulation of synaptic plasticity (ES = 1.49; *P* < 0.03), neuropeptide hormones (ES = 1.49; *P* < 0.001) and regulation of response to external stimulus (ES = 1.3; *P* < 0.006). It was interesting to note that transcription of 28 transcription factors was significantly up-regulated after REMSD (Supplementary Table S3). This suggests that REMSD sets a stage for a large scale transcriptional modulation mediated through networks of these transcription factors. Some of these transcription factors namely, BMP4, NPAS4, EGR1, EGR4, FOSB, NCOR2, PPARD and FOXO3 (Cirelli et al., 2004, 2006; Millstein et al., 2011; Elliott et al., 2014), were highly expressed upon total sleep deprivation (which includes loss of REMS as well as non-REMS). This suggests that although REMSD has several specific effects, it shares some common pathways with total sleep loss. Many genes encoding histone variants, which are involved in chromatin and nuclear assembly showed significant down regulation upon REMSD. Thus, it is tempting to speculate that the histone code and consequently the chromatin remodeling machinery are influenced by REMSD. The neuronal regulatory genes, namely, dopamine beta-hydroxylase (DBH) (*P* = 0.0009), Na–K ATPase beta-2 subunit (ATP1B2) (*P* = 0.019), synaptotagmin-2 (SYT2) (*P* = 0.011) and neurosecretory protein VGF (*P* = 0.01) showed increased expression after REMSD (Supplementary Table S2). Highlighting the altered regulatory cascades, the excitatory neuropeptide hypocretin (HCRT) (*P* = 0.0008) and synaptic plasticity related GRIN2B (*P* = 0.0006) genes were also down regulated upon REMSD.

**FIGURE 3:**
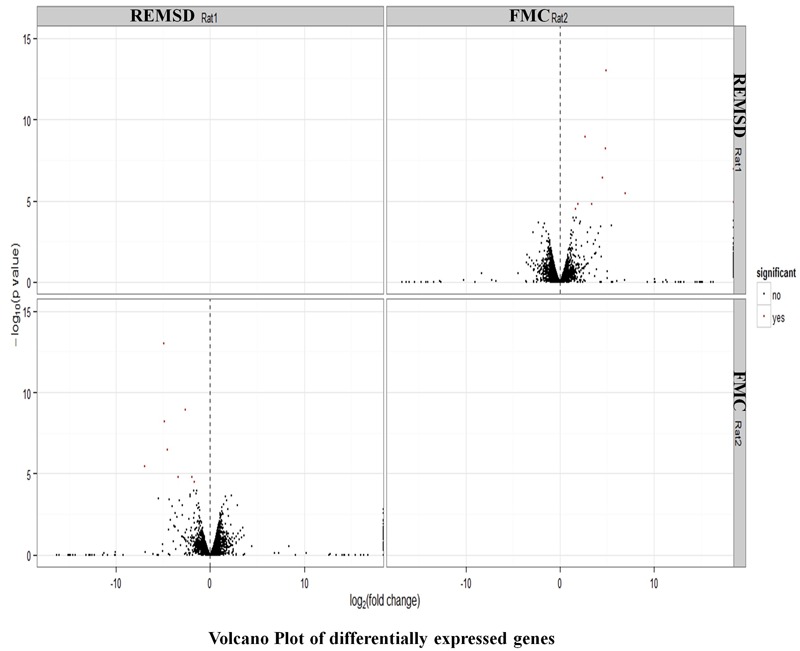
**Volcano plot of DEGs.** Volcano plot showing DEGs (*q* < 0.05), out of which 11 genes down regulated upon REMSD as compared to free moving control (FMC).

**FIGURE 4:**
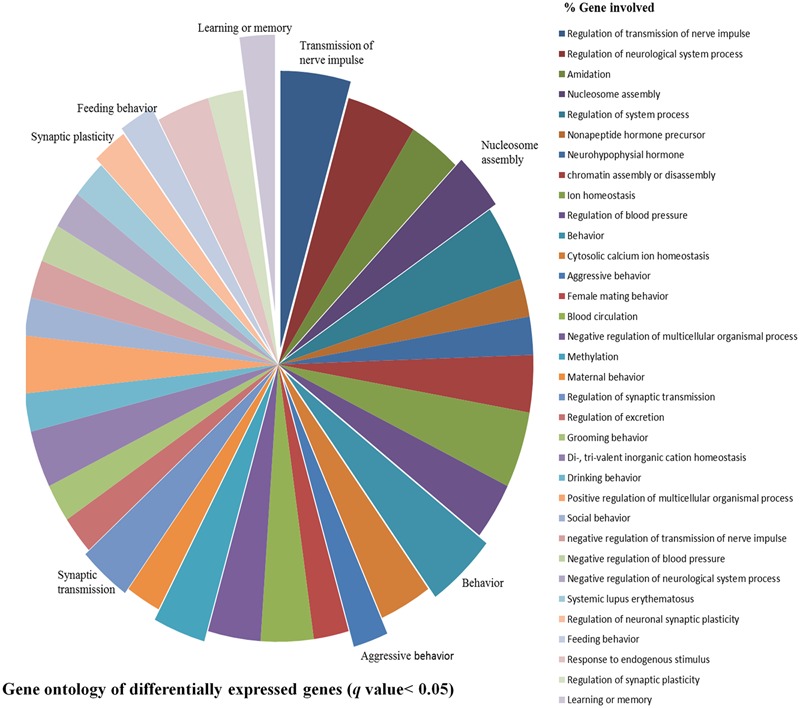
**Gene ontology of DEGs.** Gene ontology of DEGs shows association with biological processes, molecular functions and cellular component like learning or memory, nucleosome assembly, behavior, synaptic transmission, feeding behavior, synaptic plasticity, aggressive behavior and transmission of nerve impulse etc. upon REMSD.

After stringent analysis, a total of 11 genes were significantly (*q*-value < 0.05) down-regulated upon REMSD as shown in the volcano-plot **Figure 3** and Supplementary Table S4. These include oxytocin (OXT), pro-melanin-concentrating hormone (PMCH), leucyl/cystinyl aminopeptidase (LNPEP), arginine – vasopressin (AVP), hemoglobin subunit beta-1 (HBB), one long non-coding RNA–RNA component of mitochondrial RNA processing endo-ribonuclease (RMRP), one small non-coding RNA brain cytoplasmic RNA1 (BC1) and four variants of histone proteins. These gene sets were enriched under the following functional categories viz. behavior, including feeding, aggressiveness, learning and memory, nucleosome assembly, regulation of transmission of nerve impulse, chromatin assembly or disassembly and synaptic plasticity. Gene ontology of these DEGs is given in Supplementary Table S5 and gene enrichment is depicted in a pie chart (**Figure 4**).

### Validation of RNA Sequencing Data by Quantitative Real Time-PCR (qRT-PCR)

**FIGURE 5:**
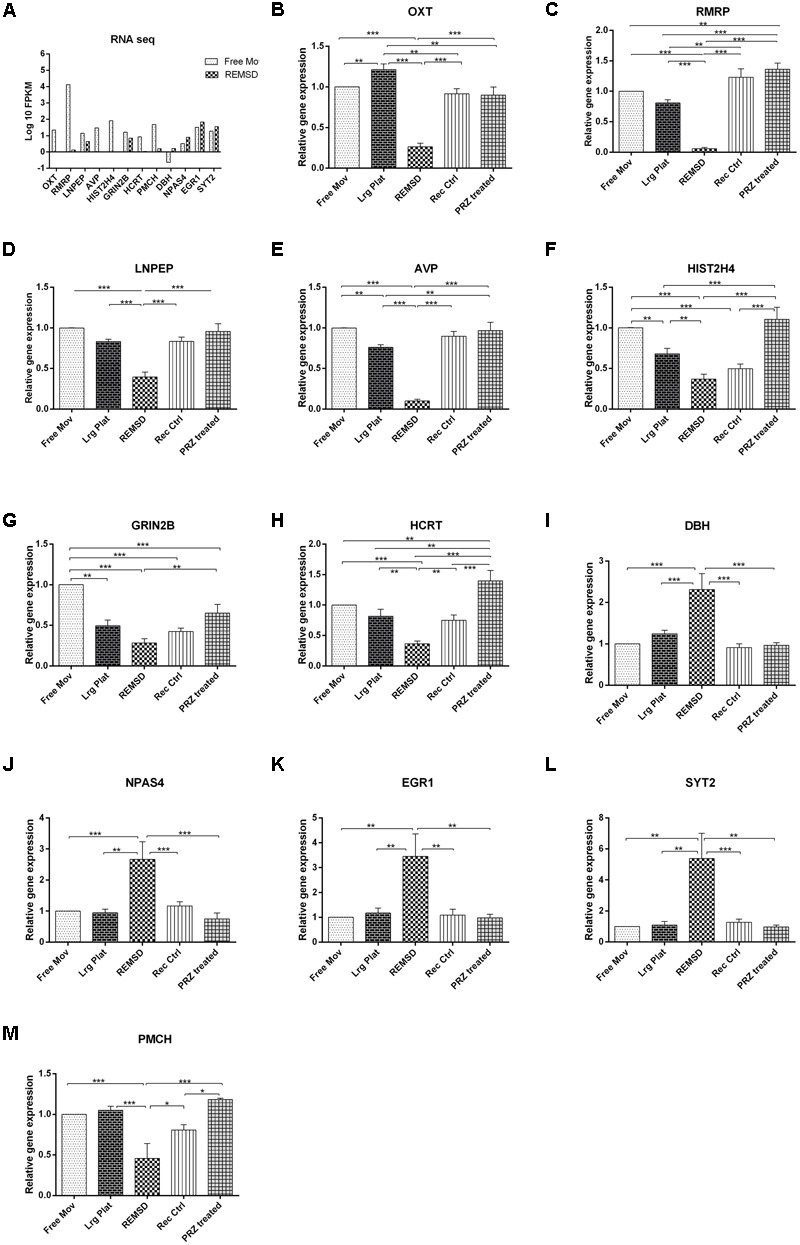
**Validation of rat brain RNA seq data using different controls.** Some genes were selected for validation of RNA seq data by qRT-PCR. **(A)** RNA sequencing results (log 10 FPKM values) showing DEGs involved in sleep and neuronal regulation like OXT, RMRP, LNPEP, AVP, HIST2H4, GRIN2B, PMCH and HCRT were down regulated upon REMSD. Other set of genes up regulated upon REMSD, which includes DBH, NPAS4, EGR1 and SYT2. For validation of these RNA seq results, we have included other additional controls like large platform control (Lrg Plat), recovery control (Rec Ctrl) and Prazosin treatment group (PRZ treated). **(B–M)** Shows qRT-PCR results for above genes in free moving control (Free Mov), large platform control (Lrg Ctrl), REMSD, recovery control (Rec Ctrl), Prazosin treatment group (PRZ treated) (*n* = 5 per group). Statistical analyses were performed using one-way ANOVA followed by Newman–Keuls multiple comparison tests. Data are expressed as the mean ± SEM (^∗^*P* < 0.05, ^∗∗^*P* < 0.005, ^∗∗∗^*P* < 0.001).

In order to validate the RNA sequencing data, 13 genes viz. OXT, LNPEP, HIST2H4, AVP, HCRT, RMRP, ATP1B2, GRIN2B, DBH, NPAS4, EGR1, SYT2 and PMCH involved in the regulation of factors controlling sleep, social memory and neuronal excitability were chosen for qRT-PCR analysis. Additional controls such as large platform control, recovery control and Prazosin treated rats were also included in our analysis. We observed that expression of mRNA of OXT, LNPEP, HIST2H4, AVP, HCRT, RMRP and PMCH genes significantly (*P* < 0.001) decreased after REMSD as compared to free moving control, large platform control, recovery control and Prazosin treated samples. The ATP1B2, DBH, NPAS4, EGR1 and SYT2 gene expression was significantly increased after REMSD as compared with other controls. Real time data and RNA sequencing data (log 10 FPKM values) of the selected genes have been summarized in **Figure 5**.

## Discussion

Rapid eye movement sleep is an essential component of sleep in mammals and in many vertebrates and its regulation is very complex. Throughout life an individual spends a significant proportion of time in REMS. It has been implicated in cognitive and higher brain functions of higher mammals including humans. Although it is expressed throughout life and its quantity is reduced with aging, it is never absent in life. The strategic importance of REMS in maintaining physio-behavioral processes may be highlighted by the fact that its loss has been shown to affect brain neurotransmitter levels and molecules regulating their synthesis and metabolism, leading to immediate as well as sustained patho-physio-behavioral changes (Kushida, 2004). Many disorders lead to disturbances in REMS, including its reduction and fragmentation, which may lead to many psycho-somatic-behavioral dysfunctions, some of which are chronic in nature. Therefore, directly or indirectly, REMSD is likely to alter the gene regulation that may be the underlying cause for the REMSD associated complex patho-physiological changes, particularly those associated to sustained changes. Although microarray analysis has been employed in earlier studies where evaluation was done after short- and long-term total sleep deprivation (which includes REMSD) of the subjects (Cirelli and Tononi, 2000; Maret et al., 2007; Massart et al., 2014), there was no report about transcriptome modulation after selective REMSD in the brain. In order to examine the changes in the gene expression pattern, we have employed NGS based transcriptome analysis of rat brain after 96 h of selective REMSD in comparison with free moving control. The expression of 216 genes involved in several physiological processes were significantly altered (**Figure 2**). This suggests that REMSD is a strong modulator of the transcriptome in the brain. Functional analysis of DEGs revealed that the expression of genes related to neurotransmission, synaptic plasticity, transcription factors, epigenetic regulators like histone proteins and non-coding RNA were significantly affected.

### Neuropeptides are Down Regulated after REMSD

We observed that REMSD significantly down-regulated expression of genes encoding hypothalamic neuropeptides like AVP, LNPEP, OXT, HCRT and PMCH in the rat brain and most of these have been shown to modulate synaptic strength (Adamantidis and de Lecea, 2009; Beets et al., 2012; Yang et al., 2013), neurobehaviors and sleep-wakefulness (Carter et al., 2012). OXT and AVP modulate social behavioral skills (Ebstein et al., 2009) and memory consolidation. LNPEP, a zinc-dependent aminopeptidase that cleaves vasopressin, oxytocin and other peptide hormones, was down-regulated upon REMSD. LNPEP is also involved in the final step of conversion of angiotensinogen to angiotensin IV and enhance acquisition, consolidation and recall in animal models of learning and memory (Stragier et al., 2008); it may have bearing with REMSD-associated hypertension. OXT is associated with cognition, tolerance, adaptation as well as complex sexual and maternal behaviors (Argiolas and Gessa, 1991) and its level elevated during active REMS (Blagrove et al., 2012). Interestingly, while dysregulated OXT levels are implicated in autism-spectrum disorders and OXT supplementation may help patients with autism; children with autism also show reduced REMS (Tanguay et al., 1976).

Hypocretin gene encodes for hypocretin (orexin), which regulates REMS, feeding behavior, metabolism (Martin-Fardon and Boutrel, 2012), learning and memory (Yang et al., 2013) and there is differential expression of HCRT upon REMSD in discrete brain areas (Mehta et al., 2015). HCRT dysregulation leads to pathological conditions like narcolepsy (Chemelli et al., 1999) and sleep apnea (Nakamura et al., 2007). PMCH gene expression was reduced after 96 h of REMSD. The highly conserved MCH system regulates multiple brain functions in the animal kingdom. MCH-ergic neurons in the lateral hypothalamus have been implicated in the regulation of sleep-wake behavior, energy homeostasis and food intake (Modirrousta et al., 2005; Luppi et al., 2013). The MCH-ergic neurons are active during sleep and positively regulate REMS (Pelluru et al., 2013) and MCH-ergic receptors are present in high density in sleep regulating areas (Jego et al., 2013). MCH knockout mice have reduced REMS (Willie et al., 2008) and genetically inactivated MCHR1 exhibited altered sleep homeostasis (Monti et al., 2013). MCH also plays an important role in learning and memory by regulating neuronal synaptic plasticity (Rao et al., 2008). We have observed that GRIN2B gene, which codes for ionotropic glutamate receptor subunit NR2B, is down-regulated in the brain after REMSD. GRIN2B has been reported to be modulated in localized brain areas after total sleep deprivation (Cirelli et al., 2006; Xie et al., 2015). GRIN2B and other *N*-methyl-D-aspartate receptor (NMDAR) are important molecular determinants of synaptic excitability and modulate synaptic plasticity and learning ability (Riedel et al., 2003). On the other hand, sleep deprivation affects hippocampus dependent learning and memory (Graves et al., 2003). Since REMSD affects cognitive ability, it is interesting to speculate that these neuropeptides and their receptors may be major players in regulation of cognitive functions, particularly for inducing chronic effects.

### Differentially Expressed Non-coding RNAs

It was seen that expression of one lncRNA viz. RMRP and one small RNA viz. BC1 were down regulated upon REMSD (**Figure 5**). RMRP possesses RNA-dependent RNA polymerase activity that produces double-stranded RNAs, which may be processed into small interfering RNA (Rogler et al., 2014). Brain cytoplasmic RNA (BC1) acts as a part of ribonucleo-complex and is involved in the regulation of neuronal protein synthesis (Zalfa et al., 2006). BC1 has the ability to be transported throughout the axons and dendrites (Muslimov et al., 2002) and reduces initiation of mRNA translation (Wang et al., 2002). BC1 regulates translation of Dopamine D2 receptor (DRD2) (Centonze et al., 2007). DRD2 stimulation in mice lacking BC1 seems to alter expression of a small non-coding dendritically localized RNA that is supposed to play a role in DRD2 mRNA translation. Repression of BC1 leads to dysregulation of plasticity and neuronal hyper-excitability (Merlin, 2010). Decreased BC1 expression after REMSD, as observed in this study may provide a link to the increased neuronal excitability (Mallick and Singh, 2011) and reduced cognitive function (Walker and Stickgold, 2014) after REMSD.

### Expression of Histone Coding Genes is Altered after REMSD

Histone code is a major contributor to chromatin remodeling and epigenetic reprogramming. The transcriptome analysis in the present study revealed that upon REMSD a major histone signature gets affected; 12 histone coding genes were down-regulated. These histones are involved in chromatin structure, nucleosome assembly, chromosome condensation, transcriptional regulation and cell differentiation. Depletion of these histone variants leads to de-repression of several gene transcription (Gossett and Lieb, 2012). The histone variants viz. HIST1H4M, HIST1H2BL, HIST1H4B, HIST1H2AIL, HIST2H3C2, HIST3H2A, HIST3H2BB, HIST1H2AN and HIST2H4 have been suggested to be involved in systemic lupus erythematosus disease (Cozzani et al., 2014). Also, total sleep deprivation, which includes REMS loss, has been reported to predispose mice to autoimmune disease (Palma et al., 2006). Thus, the findings of this study offer possible explanation underlying REMS-loss associated molecular-genetic and epigenetic mechanism(s) of action. Earlier findings could be due to complete or partial loss of REMS. Whether exclusive non-REMS loss may affect part of the changes needs further study by designing innovative experimental approach. Additionally, the importance of this study is that it shows linkages between autoimmune diseases and REMS-loss, in particular.

### Genes Encoding Transcription Factors are Upregulated

We have observed increased expression of some transcription factors after REMSD. Over expression of bone morphogenetic protein 4 (BMP4), a transcription factor, has been shown to lead to increased synthesis of SYT2 and tyrosine hydroxylase (TH) in noradrenergic neurons (Patzke et al., 2001). TH is the rate limiting enzyme for the synthesis of NA, which is elevated upon REMSD (Basheer et al., 1998). REMSD led to increased expression of DBH gene that convert dopamine to NA in the NA synthesis pathway and synaptic fusion promoting protein, SYT2 after REMSD. SYT2 help in vesicle fusion for the release of neurotransmitter including NA in the synaptic cleft (Papke et al., 2012). Thus, elevated BMP4, DBH and SYT2 show a link to the sustained increased release of NA upon REMSD (Mallick and Singh, 2011). We have earlier reported elevated levels of phosphorylated synapsin and Na–K ATPase activity upon REMSD (Singh et al., 2012). These findings support our contention that REMSD induced elevated NA increases Na–K ATPase activity (Gulyani and Mallick, 1995) and provide explanation for sustained alteration of brain excitability after REMSD. Thus, the findings offer molecular-genetics level explanation and support our hypothesis that one of the fundamental functions of REMS is to maintain brain excitability and REMS maintains house-keeping function of the brain (Mallick and Singh, 2011).

Another activity dependent transcription factor, Neuronal PAS Domain Protein 4 (NPAS4), was up-regulated upon REMSD. NPAS4 regulates the quantity and function of inhibitory synapses by causing redistribution of these synapses in the mouse hippocampus. Brain derived neurotrophic factor (BDNF), the target gene of NPAS4 coordinates this redistribution of inhibitory synapses on the cell bodies and apical dendrites (Bloodgood et al., 2013). We also observed significantly increased expression of neurosecretory VGF gene upon REMSD. VGF gene expression is induced by increased BDNF expression (Alder et al., 2003). Earlier, it has been shown that both VGF (Cirelli et al., 2006) and BDNF (Cirelli et al., 2004) gene expression were up-regulated after total sleep loss and VGF knockout mice showed impairment of spatial memory (Lin et al., 2015). The state dependent plasticity related genes like early growth response protein 1 (EGR1) (zif-268), nerve growth factor-induced protein C (EGR4) and immediate early gene FOSB show significantly increased expression upon REMSD; on other hand FOSB null mice have less REMS (Shiromani et al., 2000). These changes correlate with up regulation of early expressing genes upon total sleep deprivation (Cirelli et al., 2004; Elliott et al., 2014). Nuclear receptor co-repressor 2 (NCOR2), which is also known to be silencing mediator of retinoid or thyroid-hormone receptors (SMRT), was up regulated after REMSD. NCOR2 has been suggested to be responsible for transcriptional regulation of REMS and wakefulness (Millstein et al., 2011). NCOR2 recruits histone deacetylase and regulates target gene expression by acting as corepressor of many transcription factors. Also, peroxisome proliferator-activated receptor gamma (PPARD) gene, which is up regulated upon REMSD, interacts with NCORs and regulates sleep (Millstein et al., 2011). FOXO3 belonging to the forked head family of transcription factors, is up-regulated after REMSD. Post-transcriptional modifications like acetylation and methylation were increased after FOXO3 activity (Driver et al., 2013). Thus, our findings support that disturbances in REMS modulate epigenetic processes associated with chronic patho-behavioral changes as proposed recently (Mehta et al., 2016).

### Modulation of Gene Expression is Due to REM Sleep Loss

We have compared relative expression of some neuropeptides, histones and lncRNA after REM sleep deprivation as compared to several control conditions like free moving control, large platform control, recovery and Prazosin treated group. OXT, LNPEP, AVP, GRIN2B, HCRT, HIST2H4 and RMRP were down regulated as compared to free moving control and large platform control; their expression returned to normal levels after 3 days recovery. However, for HIST2H4 and GRIN2B genes, 3 days recovery was not sufficient for complete recovery, which could be due to many reasons including sensitivity, predisposition, and necessity of more time for recovery. Several factors which were affected upon REMSD returned to normal levels upon Prazosin treatment. This suggests that the REMSD induced effects were mediated by NA acting on alpha1 adrenoceptors and support our contention. These factors were not affected if Prazosin was injected to REMSD rats. However, RMRP and HCRT gene expression were significantly increased in Prazosin treated rats as compared to free moving control, REMSD and large platform control rats; while GRIN2B gene expression reduced after Prazosin treatment as compared to free moving control. It is possible that expression of RMRP, HCRT and GRINB2B proteins may play role(s) in modulating other functions in addition to REMS.

Only a few studies had evaluated changes in transcriptome in the total sleep deprived brain samples. However, as total sleep deprivation includes REMSD as well as non-REMSD, it was necessary to differentiate the effects of the two sleep deprivations on brain transcriptome. As normally REMS does not appear unless sufficient non-REMS has been expressed, it is practically impossible to deprive animals exclusively of non-REMS (to reasonable extent) without depriving them of complete REMS. Thus, total sleep deprivation would have compounding as well as confounding effects because it is difficult to design adequate control experiments. Also, total sleep deprivation has cascading effects, which are more difficult to interpret. Therefore, in this study we have deprived the rats of REMS in particular and used NGS to evaluate the changes. For REMSD we used the platform method, which is most effective as well as practical choice and therefore, it has been extensively used across the globe for unbiased long-term REMSD studies in animals (Gulyani et al., 2000). Notwithstanding, like most experiments particularly for *in vivo* behavioral studies, non-specific factor(s) affecting the experimental REMSD rats on the smaller platform, also acted on the large platform control and recovery control; thus the effects were negated (Gulyani et al., 2000).

Although the whole transcriptome was analyzed on free moving control and REMSD samples while a representative set of genes were analyzed in large platform control, recovery control and Prazosin treated group. The transcriptome data was validated by carrying out qRT-PCR on DEGs (**Figure 5**). By conducting large platform control, recovery control effect due to movement restriction and hyperactivity by forced swimming were neutralized, we have repeatedly shown that REMSD-associated increased Na–K ATPase activity was due to REMSD instead of other non-specific factors (Gulyani and Mallick, 1993, 1995; Gulyani et al., 2000; Mallick et al., 2002; Singh et al., 2012). Therefore, as indirect evidence that the rats were specifically deprived of REMS on small platform, in the present study we estimated the Na–K ATPase activity as well as ATP1B2 gene expression and they follow same trend. Further, the REMSD-associated transcriptional changes showed proportional expression after qRT-PCR analysis in the REMSD sample as compared to large platform control and recovery control controls confirming our contention (**Figure 5**). All the changes were significant as compared to free moving control as well as large platform control, which rule out effects due to non-specific factors including stress. Additionally, as the changes returned or tended to return to control levels in the recovery control rats, it suggests that the effects were largely due to REMSD; however, possibly more recovery time was required for those which did not return to baseline after 3 days recovery. Although expression of some of the genes changed in the large platform control samples (as compared to free moving control), the changes in REMSD samples were significant as compared to large platform control. Notwithstanding, it is possible that expressions of some genes, e.g., GRIN2B and HIST2H4 are more sensitive to some non-specific exposure, which was not ruled out by the controls. The changes in large platform control (as compared to free moving control) could be due to marginal loss of non-REMS and/or due to changes in other non-specific associated factors, which although undesirable, sometimes is difficult to avoid or control while conducting *in vivo* behavioral studies as REMSD.

It was important to differentiate between specific responses induced by treatment, REMSD in this study, against non-specific response due to stress. Therefore, it was necessary to conduct appropriate control experiments. We carried out large platform control and recovery control experiments, which were often missing in earlier similar studies. Although elevated cortisol (humans) or corticosterone (non-humans) has been considered to signify stress, there are conflicting reports on their elevation upon REMSD. In fact, many studies did not find REMSD-associated significant elevation of cortisol in humans (Born et al., 1988) or corticosterone in rats (Porkka-Heiskanen et al., 1995). Besides, as Prazosin prevented or tended to prevent most of the REMSD-associated effects, the effects were induced by NA, which increases upon REMSD. Thus, the effects observed in this study were due to REMSD and most unlikely due to stress or other confounding factors.

## Conclusion

Transcriptome analysis revealed that genes associated with expression of NA, Na–K ATPase synthesis, synaptic potentiation, chromatin, histone code and nuclear assembly and learning and memory are differentially expressed in REMS deprived rat brain. These modulations provide an underlying molecular genetic basis for REMSD-associated physiological disturbances, neurological conditions, reduced cognitive ability and reduced learning as well as memory consolidation. Also, up-regulation of Na–K ATPase activity, BMP4, DBH and SYT2 transcription offers a molecular explanation for the sustained increase in neuronal excitability upon REMSD and supports our hypothesis of role of REMS in maintaining the excitability and house-keeping function of the brain. Circumstantial evidence and control studies strongly suggest that the effects were due to REMSD and unlikely due to non-specific factors.

## Accession Code

Fastq files were uploaded to NCBI SRA database with Accession number SRR3270972 (SRP072115) and SRR3318878 (SRP072725).

## Author Contributions

SN carried out the experimental work. SN, BM, and DD were involved in designing the study, analysis and interpretation of data and writing the MS.

## Conflict of Interest Statement

The authors declare that the research was conducted in the absence of any commercial or financial relationships that could be construed as a potential conflict of interest.
